# Editorial: Relative age effect in sports: talent identification, performance, and fair practices

**DOI:** 10.3389/fspor.2025.1748381

**Published:** 2025-12-08

**Authors:** Henrique de Oliveira Castro, Ana Filipa Silva, Vivian de Oliveira, Lucas Savassi Figueiredo

**Affiliations:** 1 Grupo de Estudos em Educação Física e Esportes, Laboratório de Análise Esportiva, Universidade Federal de Mato Grosso (GEPEFE/LAE/UFMT), Cuiabá, MT, Brazil; 2 Research Center in Sports Performance, Recreation, Innovation and Technology (SPRINT), Rio Maior, Portugal; 3 Associação Brasileira de Estudos em Psicologia do Esporte e do Exercício (ABEPEEx), Curitiba, Brazil; 4 Centro de Estudos de Cognição e Ação, Universidade Federal de Minas Gerais (CECA/UFMG), Belo Horizonte, MG, Brazil

**Keywords:** talent development, youth sport, relative age effect, developmental constraints, athlete development, sport policy, talent identification, esports

The complex pathways to elite sport are often affected by selection systems designed to identify talent, yet these very systems can inadvertently create systematic inequalities. A clear example of such a bias is the Relative Age Effect (RAE): the persistent advantage enjoyed by athletes born earlier in a selection year. The RAE raises urgent questions of fairness and efficacy in talent identification, particularly in youth sport contexts characterized by early selection and specialization (1). Understanding the mechanisms, contextual moderators, and boundary conditions of the RAE is therefore central for designing talent pathways that prioritize long-term potential over transient advantage.

Over the past four decades, research has consistently shown that RAE influences participation, identification processes, and long-term success across diverse sports and cultural contexts. As the sports community strives for more ethical and evidence-based practices, it becomes paramount to examine not only the prevalence of this phenomenon, but also the conditions under which it emerges, weakens, or disappears (2). This Special Issue advances this endeavor by offering new insights into developmental, contextual, and methodological dimensions that redefine how the RAE is conceptualized and evaluated.

The seven original articles included here collectively extend the boundaries of RAE research. They span traditional and emerging contexts, from youth team sports to esports, and illustrate how relative age interacts with key factors such as maturation, sex, motor competence, athletic profiles, and performance metrics. Employing advanced analytical approaches, including artificial intelligence models alongside traditional comparative analyses, these studies deepen our understanding of the multifactorial nature of birthdate-related biases. Below, we summarize their main conceptual contributions.

Xiao et al. and Flôres et al. underscore how motor development and performance evolve through dynamic interactions among biological, experiential, and contextual factors. Xiao et al. revealed that age-related changes in basketball performance follow nonlinear patterns shaped by the interaction between physiological processes, experience, and contextual performance variables, rather than a simple linear decline. Complementarily, Flôres et al. demonstrated that sports participation and sex exert a stronger influence on motor competence in children than the RAE, highlighting the key role of environmental and experiential factors in shaping motor development. Together, these findings reinforce the need for developmental and ecological approaches in evaluating motor performance and talent progression.

Studies by Avugos and Malul and Pereira et al. (2025) reveal substantial context dependence across competitive systems. In a comprehensive analysis of male and female athletes from Brazilian national teams over more than two decades, a significant presence of RAE was observed across all categories, although it was associated only with age category type and showed no relationship with collective performance or competition type (Pereira et al., 2025). Conversely, an investigation of players from the Israeli Premier League, including both domestic and foreign athletes, found no evidence of RAE, even when considering different cut-off dates and normative birth data. Although a larger number of athletes were born in the second quarter of the year, this difference was not statistically significant (Avugos and Malul). These contrasting results show that the RAE does not manifest uniformly across countries, competitive structures, or genders and highlight the importance of context-specific analyses in understanding its real impact on talent pathways.

The contributions by Mikulič et al. and Irid et al. focus on talent identification in sports where the RAE is traditionally prevalent. Mikulič et al. analyzed isometric strength and speed performance in elite youth soccer players across different age groups, as well as potential differences in relative isometric strength between athletes selected and not selected for the national team. The authors concluded that relying solely on physical parameters for player selection and evaluation is insufficient in contemporary soccer. Complementing these findings, Irid et al. examined how athletic profiles and the RAE influence the future success of young basketball players, indicating that early advantages may interact with broader developmental factors. Together, both studies highlight the need to revise traditional talent identification models calling for multidimensional and longitudinal evaluation frameworks capable of capturing long-term potential while reducing systemic bias.

In contrast to the prevalence of RAE observed in many traditional sports, Laxdal and Erikstad investigated whether similar patterns appear in the emerging field of professional esports. Analyzing data from more than 15,000 elite players across ten game titles, the authors found no practically meaningful differences in birth-month distribution. The cognitive and skill-based nature of esports, coupled with online competition structures and the absence of early age-based selection systems, appears to mitigate the structural mechanisms that typically generate relative age advantages in traditional sports.

Collectively, the evidence in this Special Issue highlights the need to account for the interplay among individual, task, and environmental constraints (3). This perspective supports a paradigm shift in talent development, moving from systems that privilege transient physical advantages to models that foster long-term potential and reduce structural bias. To advance in this direction, sport organizations must critically examine how the RAE manifests within their specific contexts and adapt their practices accordingly. The challenge for researchers, coaches and policymakers is to translate these insights into concrete actions by standardizing monitoring processes, broadening selection criteria, and piloting policy reforms. Through coordinated and sustained efforts, talent ecosystems may evolve toward greater fairness, efficacy, and enduring excellence.
